# Evaluation of the experience with the use of telemedicine in a home dialysis program—a qualitative and quantitative study

**DOI:** 10.1186/s12882-022-02824-5

**Published:** 2022-05-19

**Authors:** Raquel Scofano, Alexandra Monteiro, Luciana Motta

**Affiliations:** Telessaúde UERJ, Universidade do Estado do Rio de Janeiro, UERJ, Avenida Vinte Oito de setembro 77, 3° andar, Rio de Janeiro-RJ, Vila Isabel CEP 20551-030 Brazil

**Keywords:** End-stage renal disease, Home dialysis, Telemedicine, Telenephrology, Telemonitoring, e-Health

## Abstract

**Introduction:**

Assisted home hemodialysis is a therapeutic modality for patients diagnosed with end-stage renal disease who require dialysis replacement therapy and have concomitant health limitations that prevent them from attending a satellite dialysis unit or performing their own treatment.

**Objective:**

The main objective of this study was to evaluate whether telemedicine provided through telemonitoring can improve the ongoing relationship between the doctor, the nurse and the patient.

**Method:**

This prospective longitudinal, qualitative and quantitative study analyzes the impact of telemedicine through an evaluation of the experiences of patients and nurses. During the study, we performed remote weekly monitoring for 6 months.

**Results:**

A total of 17 patients and 12 nurses were included. We observed that the patients and nurses had positive experiences with telemonitoring and highlighted feelings of being cared for and improved confidence, although they indicated that telemonitoring does not replace face-to-face visits.

**Conclusion:**

Telemonitoring is a useful tool to increase satisfaction with and confidence in home hemodialysis.

## Introduction

Assisted home hemodialysis (HHD) is a hemodialysis modality performed at home by trained nurses and is indicated for patients with motor or functional limitations that prevent them from attending satellite clinics or performing their own treatment [1].

Adherence to this therapeutic modality is directly related to increased comfort at home [1, 2]. However, HHD patients have concerns about undergoing hemodialysis at home, such as feelings of social isolation, a lack of connection with medical care and doubts and fear of complications [3–5], which suggests that patients on home dialysis may need additional support to improve their satisfaction and treatment adherence [6].

Telemedicine allows remote contact between patients and health professionals to improve adherence to the hemodialysis regimen and allow patients to feel connected to health professionals [3]. In addition, telemedicine is an excellent tool for monitoring treatment, adjusting medications, answering questions and providing continuing education to patients and health professionals [7, 8].

Currently, patient experience is recognized as one of the three pillars of care quality. Empathic information, communication and respect for the patient’s beliefs can keep patients more involved in treatment. [9–11].

The objective of this study was to evaluate experiences with medical telemonitoring among patients diagnosed with chronic kidney disease who were enrolled in an assisted HHD program and among nurses who performed the treatment.

## Materials and methods

After the study protocol was submitted to and approved by the Research Ethics Committee, 17 patients and 12 nurses were selected. Physicians, social workers and dieticians were not evaluated. Patients who had been in the HHD program for at least 3 months, were aged 18 years or older and agreed to answer the evaluation questionnaire were included. Patients who did not have internet access or had a poor internet signal, patients who were clinically unable to interact with the doctor during telemonitoring and nurses who were removed from the program for any reason were excluded. All participants provided written informed consent to participate in the study.

Telemonitoring was performed from June 2, 2020, to January 20, 2021, which corresponded to 6 months of monitoring. The investigator was responsible for all online consultations throughout the study period. Weekly telemonitoring was performed with each patient individually for an average duration of 20 min. Telemonitoring was performed during hemodialysis. Only one patient requested that hemodialysis be performed outside of this period because he liked to sleep during the treatment. During telemonitoring, we used the ZOOM platform because it is easy to handle and allowed recording of all interviews.

In total, 280 interviews were conducted during the study period. The interviews were digitally recorded and transcribed by the researchers.

### Application of the evaluation questionnaire

Qualitative and quantitative methods were chosen to allow an exploratory analysis because knowledge on the subject is limited. Semistructured interviews were used to deepen the understanding of the individual experiences of the patients and nurses.

For the quantitative evaluation, we selected two complementary instruments after adaptation and translation for a broad exploration of the patients’ and nurses’ experiences with the telemonitoring program. The first study by Whitten et al. [12] was selected because of its proximity to the profile of the present study’s population. This instrument assesses the perception of the use of telemedicine in patients receiving remote care from nephrologists during hemodialysis. The second instrument was the Acceptability E-scale (AES), which is a generic and validated measure developed by Tarimn et al [13] to measure the acceptability and usability of e-Health systems.

The qualitative evaluation used an interview script for which the main question was “What would you tell a friend about your experience with telemonitoring?” The interview question was open, and participants were encouraged to provide examples and expand on their responses.

The results of the quantitative component were analyzed using descriptive statistics for the sociodemographic and clinical variables and for the results of the questionnaire, which is composed of closed questions and Likert-scale responses. The qualitative component included text corpora and content analysis of the answers to an open-ended question using the focus group technique and the thematic-categorical content analysis method developed by Bardin [14].

## Results

A total of 17 patients and 12 nurses were selected. The study patients had a mean age of 80 years, and most were male, white and had completed higher education. All patients have family members or caregivers who help with daily care. Their mean time on HHD was 3.2 years. The main cause of renal function loss was hypertension, followed by diabetes (see Table 1).

**Table 1:** Patient characteristics in the study conducted to evaluate experiences with the use of telemedicine in a home dialysis program

Variable	N (%) or Mean ± SD
Patients	17
Age (years)	80 ± 20
Male	64%
Race/ethnicity	
White	85%
Black	10%
Other	5%
Higher education	76%
Mean time on HHD (years)	3.2 ± 1
Dialysis frequency per week	
2 sessions	76%
3 sessions	12%
6 sessions	12%
Main causes of renal function loss	
Hypertension	41%
Diabetes	23%
Glomerulopathy	12%
Uropathologies	11%
Other	13%
Index of Coexisting Disease	
Severe	82%
Moderate	6%
Mild	12%

*Abbreviation: SD* Standard deviation

The HHD patients in this sample had a large number of comorbidities, and 82% had more than three comorbidities. When the Index of Coexisting Disease (ICED) was applied, 82% were classified as severe, 6% as moderate and 12% as mild. Each patient used a mean of 9 medications continuously.

Most hemodialysis sessions were performed three times a week and lasted 4 h. Only two patients underwent daily dialysis to aid in the treatment of heart failure and volume management. Two other patients were selected for incremental hemodialysis twice a week because they had good residual urine output.

The sociodemographic data of the nurses showed a mean age of 43 years; 76% were women, and 70% were white. They had a mean duration of professional experience of 5.5 years (see Table 2).

**Table 2:** Patient characteristics in the study conducted to evaluate experiences with the use of telemedicine in a home dialysis program

Variable	N (%) or Mean ± SD
Nurses	12
Age (years)	43 ± 5
Women	76%
Race/ethnicity	
White	70%
Black	15%
Other	15%

*Abbreviation*: *SD* Standard deviation

### Evaluation of patient experience

To analyze the data more broadly, we divided the responses into three distinct points: the ease of use of the technology, the quality of the synchronous telemonitoring visits, and comparisons with face-to-face visits. The answers to the open question are organized in Tables 3 and 4.

**Table 3:** Themes, subthemes and patients’ answers to the open questions in the study conducted to evaluate their experience with the use of telemedicine in a home dialysis program

Theme	Subtheme	Answer
Ease of use of the technology	Patient responses regarding the ease of using the communication technology	P1: *“It was easy to use the technology; the nurse did not need to help me. In telemonitoring, the patient can have contact with the doctor every day, if necessary.”* P8: *“It was easier to talk to the doctor, and I was able to have questions answered and report problems faster. Additionally, I found it interesting to use the technology for hemodialysis treatment.”*
	Patient responses regarding the ease of using telemonitoring	P3: *“I liked it a lot because we have questions answered and we are supported by the doctor’s guidance.”* P5: *“We have little contact with the doctor, so it’s easier to talk to the doctor, since we have little contact with the doctor on a daily basis. It was important to have questions answered when necessary.”*
	Patient responses regarding the time spent on telemonitoring	P1: *“I think I should continue with telemonitoring even after the study. I liked it very much.”* P3: *“I think the video call should take place in every dialysis session and not just once a week.”*
Quality of the synchronous telemonitoring visits	Patient responses regarding the quality of care through telemonitoring	P1: *“The experience was very good because I felt the interest in me. I think it’s worth it because it gives the patient confidence in the work being done. I felt the interest of the professionals who take care of my health, and I feel safer.”* P2: *“However, I liked having a doctor regularly wanting to know about my treatment; that was good.”* P6: *“The experience was good. You feel more cared for; things are more under control, and some gaps end up being filled. I thought it was a good experience.”*
Comparisons with face-to-face visits		P1: *“Telemonitoring is the future form of medical care. The in-person presence of the doctor can generate excessive costs”* P6: *“Telemonitoring is something feasible. It works very well for maintenance treatment, especially in chronic kidney patients, who already know the doctor, who already have an established therapy; it adds a lot.”*

**Table 4:** Themes, subthemes and nurses’ answers to the open questions in the study conducted to evaluate their experience with the use of telemedicine in a home dialysis program

Theme	Subtheme	Answer
Ease of use of the technology	Nurses’ responses regarding the ease of using the telemonitoring technology	N1: *“It was a new thing. I was anxious, nervous, didn’t know how to explain it to the patients, didn’t know if they’d like it. In my case, the patients always wanted to talk to the doctor, but I felt watched….”* N2: “*A sensational, simple thing. It was the first time I did it, and I liked it. It will greatly change the form of care.”*
	Nurses’ response regarding the ease of communicating with the doctor	N2: *“With the weekly monitoring, I felt more confident. It was complementary. The doctor was with me the whole time, giving instructions, providing assistance.”*
Quality of the synchronous telemonitoring visits	Nurses’ responses regarding care quality during telemonitoring	N3: *“I thought it was a seven-headed monster, but it was easy. The visit was short and objective, and the questions asked (by the patients) were answered. The patients needed it; it gave them confidence.”* N2: *“…something new is coming. It gives more confidence to the staff and patients. I liked it very much.”*
Comparisons with face-to-face visits	Nurses’ responses regarding the comparison of telemonitoring with face-to-face doctor visits	N4: *“Seeing the doctor once a month is too little. Telemedicine came as a complement for providing quality care.”* N6: *“Good experience, very helpful, I liked it, I found it very appropriate, but I think the face-to-face visit with the doctor is very important.”*

Although the patients in this sample were elderly, we found that they had an easy time adjusting to the use of telemonitoring technology, as cited in Table 3.

**Fig. 1:**
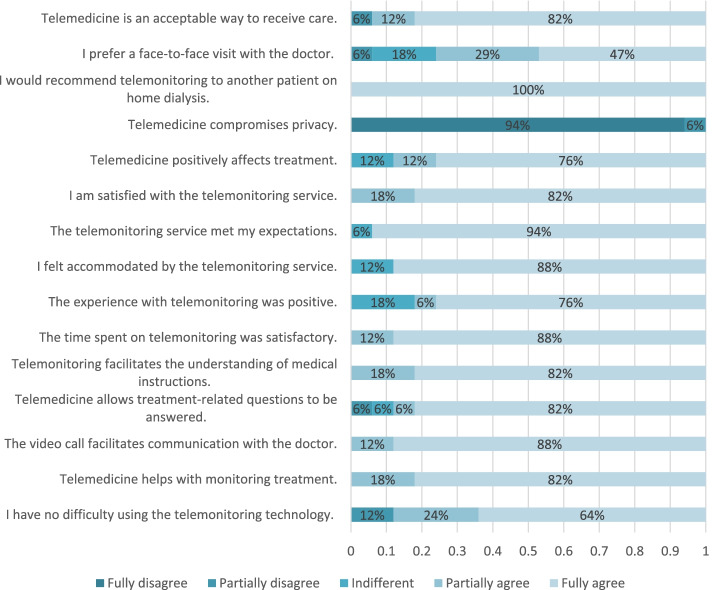
Results of the questionnaire evaluating the patients’ experiences with telemonitoring

In response to the questionnaire “I have no difficulty using telemonitoring technology”, 64% totally agreed, 24% partially agreed, and 12% partially disagreed, as shown in Fig. 1. The greatest difficulty that the patients cited in using the technology was a low data transmission speed, which prevented or reduced the quality of communication.

Most of the patients fully or partially agreed that telemonitoring helps in monitoring their treatment, increases communication with the doctor and increases their understanding of medical instructions.

Regarding whether the time spent on telemonitoring was sufficient, 88% fully agreed and 12% partially agreed. During the answer to the open question, patients reported that they would like telemonitoring to be carried out more frequently.

Although the technology had good acceptability among the patients undergoing HHD, patients’ opinions regarding how telemonitoring impacts treatment and their daily lives must be understood. Patients generally found the quality of telemonitoring to be positive.

When patients were presented with “The experience with telemonitoring was positive”, 76% fully agreed, 18% were indifferent, and 6% partially agreed. For the statement “Telemedicine positively affects treatment”, 76% fully agreed, 12% partially agreed, and 12% were indifferent. Regarding satisfaction, 82% fully agreed and 18% partially agreed that they were satisfied with telemonitoring.

Regarding the statement “I felt accommodated by the telemonitoring service”, 88% fully agreed, and 12% were indifferent.

Telemonitoring did not seem to significantly compromise patient privacy. A total of 94% of the patients strongly disagreed with the statement “Telemonitoring compromises privacy”. Only one patient partially disagreed.

Although telemonitoring had a different impact on each individual, 88% fully agreed that the video call facilitated communication with the doctor, and all of the patients fully agreed that they would recommend the service and telemonitoring to other HHD patients.

Face-to-face visits with the doctor were considered a more complete form of care due to the possibility of undergoing a physical examination. For the question “I prefer a face-to-face visit with the doctor”, 47% fully agreed, 29% partially agreed, 18% were indifferent, and 6% partially disagreed. Nevertheless, the patients considered telemedicine a complement to medical care with good acceptability, especially during the pandemic period. Regarding the statement “Telemedicine is an acceptable way to receive care”, 82% fully agreed, 12% partially agreed, and 6% partially disagreed. Regarding answers to the open question, patients considered telemonitoring to be the future form of medical care.

### Evaluation of the nurses’ experience

At the beginning of the study, we observed a lack of confidence among some nurses regarding the use of the technology, mainly because they felt that they were unable to use the platform; however, after the initial training, they overcame this barrier and were able to use the equipment easily, as cited in Table 4.

**Fig. 2:**
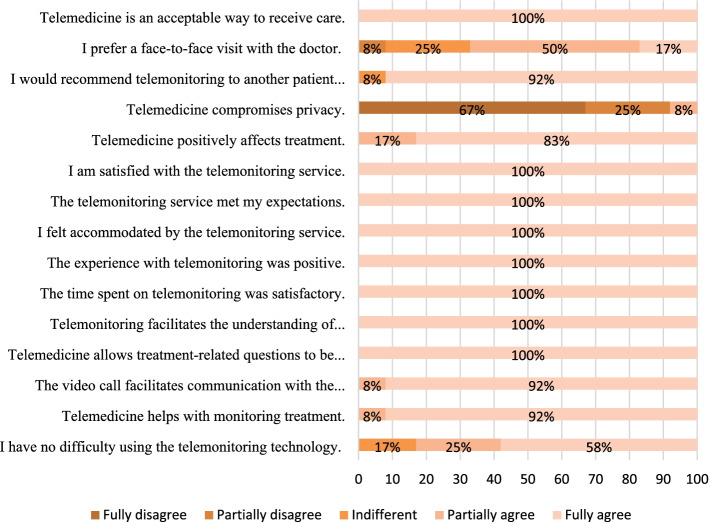
Results of the questionnaire evaluating the nurses’ experience with telemonitoring

Regarding the statement “I have no difficulty using telemonitoring technology”, 58% fully agreed, 25% partially agreed, and 17% were indifferent, as shown in Fig. 2. After 6 months of use, they considered telemedicine an innovative and useful tool, as cited in Table 4.

The statements “The experience with telemonitoring was positive”, “The telemonitoring service met my expectations”, “I am satisfied with the telemonitoring service” and “I felt accommodated by the telemonitoring service” received 100% total agreement. This positive response may be explained by the increased interaction with and availability of the doctor that both the patient and the nurse experienced during the daily routine of HHD.

The nurses reported a positive experience in clarifying their doubts, as well as helping with decisions at critical moments.

When asked about “Telemedicine compromises privacy”, 67% of the nurses completely disagreed, 25% partially disagreed, and 8% partially agreed. At the beginning of telemonitoring, many of the nurses felt they were being monitored, but with time and an increase in empathy, they began to view telemedicine as an ally in their daily practice. Regarding the answers about “I would recommend telemonitoring to another patient on home dialysis”, 92% fully agreed, and 8% were indifferent.

The nurses easily accepted the guidelines given by video calls. The statement “Telemedicine is an acceptable way to receive care” received 100% total agreement. Regarding “I prefer a face-to-face visit with the doctor”, 17% fully agreed, 50% partially agreed, 25% were indifferent, and 8% fully disagreed.

### Textual analysis

**Fig. 3:**
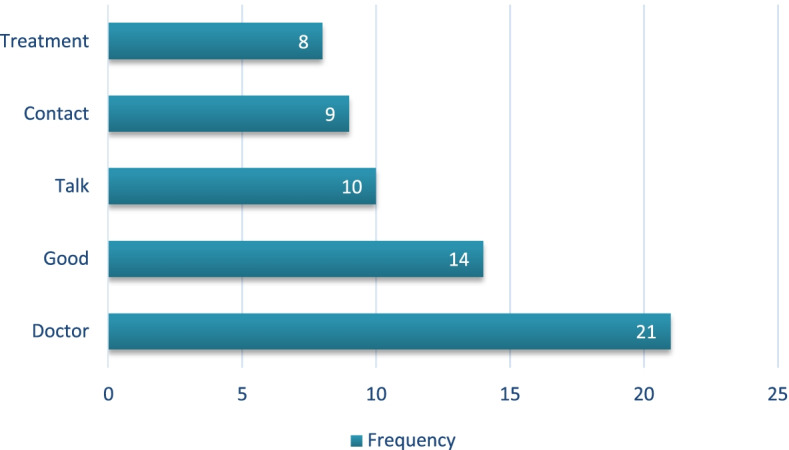
Absolute frequency of the words in the analysis of patients’ text corpora

An analysis of 29 text corpora was performed. We observed that the words “doctor”, “good” and “talk” were the top 10 most frequently used words, reflecting the impact of telemonitoring on increasing the patients’ and nurses’ contact with the doctor, as represented in Fig. 3.

**Fig. 4:**
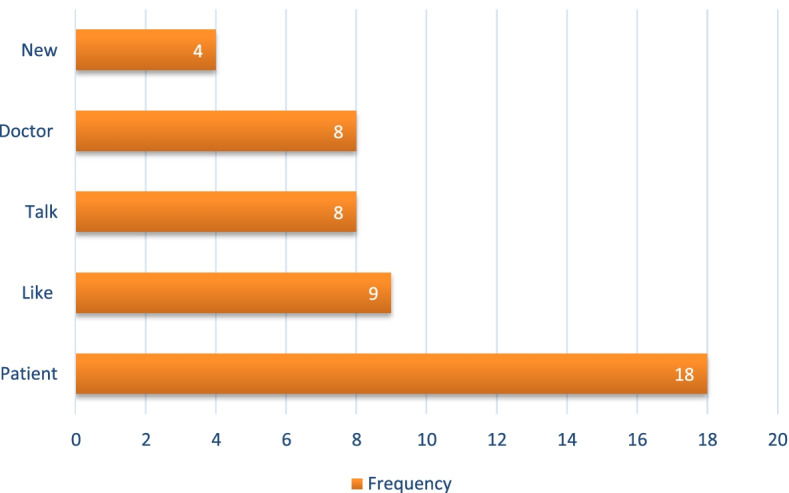
Absolute frequency of the words in the analysis of nurses’ text corpora

In the textual analysis of the nurses’ responses, the word “patient” was the most frequently used, representing concern and patient-centered care, as represented in Fig. 4.

## Discussion

Our study showed that elderly patients with comorbidities who were on HHD accepted telemonitoring but would not give up receiving face-to-face medical care whenever possible. The nurses who performed HHD, despite initial resistance to remote medical supervision, also provided positive evaluations of telemonitoring.

Many studies have described the use of telemedicine as a form of intervention for patients with chronic kidney disease according to the systematic review by Shen et al. [15], but only three studies specifically referred to home dialysis.

Home dialysis combined with telemedicine was considered one of the main tools for supporting distancing and was considered a public health measure by some countries, reflecting Truong et al.’s [16] study regarding new policies for dialysis treatment in the United States. No randomized study on the use of telemedicine by patients undergoing HHD has been conducted to date.

This study evaluated the experience of patients and nurses enrolled in an HHD program with synchronous telemonitoring performed by a physician for 6 months.

In a pioneering manner, our study addresses a population with several comorbidities and motor limitations that hinder their ability to travel to hemodialysis centers and receive help from nurses for home treatment. In contrast, previously published studies, such as that of Liu et al. [3], portray HHD as a form of self-care or a means of overcoming geographical barriers, reflecting the uniqueness of our study.

The HEMO study applied the ICED to a population of patients undergoing dialysis at a satellite clinic to evaluate their profile in terms of the risk of hospitalization and morbidity [17]. The distributions of normal, mild, moderate and severe patients according to the ICED were 0.2%, 34.9%, 31.2% and 33.7%, respectively. In our study population, the high severity index of 82% stood out, indicating the frailty of our patients.

To evaluate patients’ experience with telemonitoring, we divided the analysis into three main points: the ease of technology use, the quality of synchronous telemonitoring and comparison of synchronous telemonitoring with face-to-face visits.

Although the population comprised elderly individuals, they had no difficulty using the technology. Diamantidis et al. [18] in 2015 used an application to assist with medication use in a patient population similar to that in our study and described that their population, although elderly, made regular use of the technology at home through laptops and had no difficulty with it.

At the beginning of telemonitoring, the nurses felt a lack of confidence and distrust associated with a feeling of a loss of privacy due to the evaluation, but they soon adjusted to the technology and began viewing it as an ally in their work. These professionals were instrumental in the implementation of telemonitoring as device facilitators and drivers of adherence. In 2021, during the period of the COVID-19 pandemic, the American Society of Nephrology COVID-19 Home Dialysis Subcommittee published incentive measures and guidelines for the use of telemedicine in which nurses are described as having a prominent role in the implementation of this technology [19].

When we evaluated the quality of care provided by videoconferencing, the patients reported feelings of insecurity due to the distance from the doctor during the HHD procedure, and an increased need for day-to-day care for a population with multiple comorbidities was evident. The patients reported having a positive experience, a high degree of satisfaction and a sense that the intervention was favorable for their treatment based on the greater sense of confidence and accommodation that telemonitoring provided them. They were able to have their treatment-related questions answered and obtain more detailed information on diet and medication use. In addition, empathy and individual-focused care helped the patients overcome the anguish caused by social distancing without negatively affecting their privacy. The increased frequency of medical consultations can identify complications earlier, thereby preventing exacerbations, and can provide specific guidance for care, thus generating trust, which also increases the safety of the nurses’ work. These data are consistent with the available literature. Nadeau-Fredette et al. [20] conducted a multicenter study in Canada in which several potential complications during home dialysis were identified through telemedicine, and patients were satisfied with the care that they received [20]. Review studies on telemedicine and patient satisfaction showed increased satisfaction with the use of technology for monitoring, which can have a positive impact on the entire treatment by improving patients’ engagement with their treatment, their health and their quality of life [3, 21]. Liu et al. [3] evaluated the experiences of patients and nurses in Australia with remote monitoring using safety alarms for clinical parameters, which were sent to nurses over a 128-day monitoring period. As in our study, both the patients and nurses had positive experiences, and the major positive points were the saving of time previously spent commuting, greater empathy and consequent adherence to treatment.

When comparing care provided via video calls with face-to-face visits with the doctor, we found that telemedicine was acceptable to both the patients and the nurses, especially during the pandemic, but was considered complementary to face-to-face visits. Its main limitation was the lack of physical examinations. Walker et al. [22] conducted a systematic review of patients’ telemedicine for monitoring chronic diseases. For patients with chronic diseases, remote monitoring increased their disease-specific knowledge, triggered earlier clinical assessment and treatment and improved self-management and shared decision-making. However, these potential benefits were balanced against concerns about losing interpersonal contact and the additional personal responsibility associated with remote monitoring [22].

## Conclusion

The patients’ and health professionals’ experiences with telemedicine were positive mainly because it increased their confidence and sense of care during HHD. The initial resistance of nurses was probably greater than that of patients due to the possibility of evaluating their care performance, creating a feeling of discomfort. Telemedicine was deemed an acceptable way to receive health care but was regarded as complementary to face-to-face visits with the doctor. Telemedicine could be considered a valuable tool for coping with distancing while providing HHD, and health professionals expressed their confidence in and acceptance of this delivery method.

Further studies are needed to show the impact of telemedicine on physicians, nurses, social workers and dieticians participating in an HHD program and possible correlations with clinical and laboratory parameters.

## Data Availability

The data analyzed during this study are included in this published article.

## Abbreviations

HHD: Home hemodialysis
AES: Acceptability E-scale
ICED: Index of Coexisting Disease
